# Are Child Domestic Workers Worse Off than Their Peers? Comparing Children in Domestic Work, Child Marriage, and Kinship Care with Biological Children of Household Heads: Evidence from Zimbabwe

**DOI:** 10.3390/ijerph19127405

**Published:** 2022-06-16

**Authors:** Ronald Musizvingoza, Jonathan Blagbrough, Nicola Suyin Pocock

**Affiliations:** 1 United Nations University International Institute for Global Health (UNU-IIGH), Kuala Lumpur 56000, Malaysia; 2 Children Unite, London E9 7JR, UK; jonathan.blagbrough@childrenunite.org.uk; 3 Department of Global Health and Development, London School of Hygiene and Tropical Medicine, London WC1E 7HT, UK; nicola.pocock@lshtm.ac.uk

**Keywords:** child domestic work, child labour, hazardous work, child marriage, kinship care, Zimbabwe

## Abstract

Child domestic work is a hidden form of child labour driven by poverty and social norms. However, little is known about the situations of child domestic workers. This study aims to describe and analyse gender-specific working conditions, health, and educational outcomes among hidden child domestic workers (CDWs) living in third-party homes relative to married children, biological children, and other children in kinship care. Data from the 2019 Zimbabwe Multiple Indicator Cluster Survey (MICS) were analysed. Descriptive statistics and bivariable logistic regression were used to describe frequency and estimated prevalence. Directed Acyclic Graphs (DAGs) were used to identify exposures and inform the selection of covariates. Multivariable logistic regression models were fitted to estimate the effect of each exposure variable. The prevalence of CDWs was 1.5% and CDWs were mainly girls and living in much wealthier households with more educated household heads while married girls were living in much poorer households. When compared among girls themselves, being a CDW was significantly associated with having a functional disability, while married girls were more frequently engaged in hazardous working conditions. We provide the first intersectional analysis comparing work, violence, and health outcomes among CDWs, married children and other children. Child protection measures are needed to safeguard children in domestic work and marriages.

## 1. Introduction

The widespread use of children as domestic workers (CDWs) is one of the most hidden forms of child labour [1]. Globally, an estimated 17.2 million children are in paid or unpaid domestic work in the home of a third party or employer. Of these, 11.5 million are in child labour, of which 3.7 million are in hazardous work which constitutes 21.4% of all CDWs [2]. More children might be in child labour since the global estimates significantly underestimate the prevalence due to their reliance on household surveys that do not accurately capture all children in child labour [3]. A CDW refers to children’s work in the domestic work sector in the home of a third party or employer. CDWs may be living and working with distant or close relatives (e.g., aunts and uncles) and working under “fostering” type arrangements in the home of a third party [3,4]. CDWs are often hidden and hard to tackle because of their links to social and cultural patterns which tolerate child labour and encourage some forms of alternative care that expose children to child labour. In many countries, CDWs are not only accepted socially and culturally but are also regarded in a positive light as a protected and non-stigmatised type of work as well as being preferred to other forms of employment—especially for girls.

Child marriage, defined as any formal marriage or informal union where at least one party is aged below 18, affects an estimated 12 million girls every year [5], with an estimated 19% of girls married by age 18 (ibid). Approximately one in ten children globally are in kinship care [6]. Analysis of nationally representative household surveys in 77 countries showed that the vast majority of children aged below 15 not living with either biological parent were residing with relatives (86.7% in the Gambia to 99.5% in Togo) [7]. Kinship care is defined as a form of alternative, family-based care within the child’s extended family or with close family friends, whether formal or informal by nature, in the United Nations Guidelines for the Alternative Care of children [8]. Children may reside with relatives, godparents, stepparents, or non-relatives in their extended community.

Despite potential similarities between CDWs and married children, and between CDWs and children in kinship care, no research explores the intersections between children in these varying situations or the overlapping drivers and motivations for selection into these situations. For example, being a CDW can be seen as suitable preparation for early marriage in contexts where it is acceptable for girls to migrate alone [9,10]. Being a CDW can also be a preferred route to postpone or avoid marriage among girls, such as in Nepal [11] and Togo [12]. When it is socially unacceptable for girls to migrate alone, they may marry early to migrate, then end up working as a CDW at the destination, as documented in Ethiopia [13]. Early marriage can also act as a guise for girls being unpaid CDWs in their husbands’ relatives’ homes, alongside the marital home [14]. Overall, both being a CDW and early marriage are considered safe or preferred options for out-of-school girls by families, being traditionally protective mechanisms including reducing the likelihood of pre-marital pregnancy and alleviating the economic burden on families [3].

Being a CDW is associated with various hazards linked to denial of rights which can have irreversible physical and psychological impacts on health and wellbeing, such as access to education and health care, and the right to rest, leisure, play, and recreation. A recent systematic review found a large variation in experiences of violence among CDWs in Low and Middle Income Countries (LMICs), with over half of CDWs experiencing emotional violence (56%), and 19% and 2% experiencing physical and sexual violence, respectively (median rates) [15]. Across studies, between 7% and 68% of CDWs reported work-related illness and injuries, and one third to half had received no medical treatment. On average, children worked between 9 and 15 h per day with no rest days [15]. Child marriage is also strongly associated with violence and poor health outcomes, including early pregnancy, maternal and child morbidity and mortality, and intimate partner violence (IPV) [16]. Nearly a third of young women who married as children experienced IPV in the past year compared to a fifth of women who married as adults [17]. Broadly, there are many more studies of interventions for child marriage than for CDWs. Recent systematic reviews have found just one CDW targeted intervention among five evaluations, compared with 30 quasi-experimental and experimental evaluations targeting child brides [16,18]. There are no studies on disability among CDW, and among child brides, there is insufficient evidence on whether children with disabilities are more or less at risk than those without disabilities [19].

Similarly, overlaps between CDWs and children in kinship care have not been considered in research. Evidence has shown that, compared with children living with one or both parents, children in nonparental care, including kinship care, are in poorer health, are at heightened risk for experiencing disruptions and instability in caregiving and are vulnerable to other social antecedents of child health (e.g., neglect, poverty, maltreatment [20]. There is limited research on the impact of disability on kinship care [6]. Since CDWs are living in nonparental care and engaged in labour activities they might be exposed to worse conditions when compared to non-CDWs, including children in kinship care who are not undertaking an undue burden of work. It is also unclear how differential treatment of children in households affects wellbeing outcomes. A rare example is a qualitative study in Tanzania that compared experiences and wellbeing of CDWs with employers’ children (i.e., directly related sons and daughters) [21]. CDWs experienced emotional and material benefits from good relations with biologically related employers’ children, which included resisting adult control over their mobility. The research highlighted the unique position of employers’ children for potential intervention programming in schools, to create household environments sensitive to CDW needs. Furthermore, CDWs preferred not to work for relatives (in de facto kinship care situations) as this often meant they were not paid (or not paid on time), and mistreatment by relatives felt more painful than when it was experienced in non-relative households (ibid). Living arrangements are clearly a determinant of both children’s experience of work and wellbeing yet are rarely considered simultaneously.

A major challenge for research into these overlapping phenomena is that children in these categories remain hidden or simply are not disaggregated in child health studies. In particular, many CDWs are likely to remain hidden due to underreporting of CDWs for reasons including employers not considering CDW as employment, reporting unpaid household chores only in household surveys, and deliberate concealment of CDWs in exploitative situations [4]. CDWs can also be misreported as fostered or adopted children doing household chores [22,23]. However, household surveys such as the MICS can be used to estimate the prevalence and explore the conditions of CDWs alongside other groups of children.

This study aims to describe and analyse gender-specific working conditions, health, and violence outcomes among hidden CDWs living in third-party homes relative to married children, biological children, and other children in kinship care. The objectives are to:

1. Describe the prevalence of hidden CDWs by examining hours spent on household chores and relationship to the head of household (niece, nephew, distant or another relative);
2. Explore prevalence of Child Labour among hidden CDWs and other categories of children.
3. Compare working conditions, including occupational hazards and maltreatment, among hidden CDWs and other categories of children.
4. Analyse health outcomes of hidden CDWs and other categories of children.

## 2. Materials and Methods

### 2.1. Data

We used data from the 2019 MICS in Zimbabwe, a nationally representative sample survey. The survey was carried out by the Zimbabwe National Statistics Agency, as part of the Global MICS Programme. The survey provides internationally comparable demographic and health data on a wide range of indicators about children and women for policy and programmatic purposes such as monitoring progress in sustainable development indicators. Specific topics covered in the survey include information related to children’s well-being, women, and households, ranging from health and education to child protection and water and sanitation. The survey design of the MICS is a stratified two-stage cluster sampling method based on census enumeration areas (EAs) and household samples. Urban and rural areas within each province were identified as the main sampling strata and the sample of households was selected in two stages. The first stage was the selection of EAs with probability proportional to the size. The second stage involved household selection, where households were selected based on the EAs using systematic sampling. As a result, a total of 462 clusters and 12,012 households were selected at the national level.

The analysis was limited to children aged 5–17 years with complete information on their relationship and child labour status. The module for children aged 5–17, which contains information on the child’s background, child labour, child discipline, child functioning, parental involvement, and foundational learning skills, was retrieved for data analysis. This dataset was linked to the household dataset to determine the relationship to the household head and other sociodemographic characteristics of the child, mother, and household. Using this information, we can explore the prevalence of hidden CDWs who are usually living as other relatives in the households and explore their living conditions, as well as compare their situation relative to married children, biological children, and children in kinship care. Children with invalid or missing information such as age and male children listed as household heads, spouses, and sons-in-law were omitted from the study. As a result, the analysis was limited to 15,023 children aged 5–17 years with valid relationship status and child labour information.

### 2.2. Variables

#### 2.2.1. Categories of Children

##### Child Domestic Workers

To categorise CDWs, we used the household roster to first determine children living in third-party households who were likely to constitute ‘hidden’ CDWs when combined with a threshold of hours spent on household chores, as per recommendations for detecting hidden CDWs [4]. In this study, 21 h or more was the selected threshold for hours spent on household chores across all age groups 5–17. Twenty-one hours correspond with the child labour (household chores) classifications by the International Labour Organisation (ILO), with the exception that we have applied this threshold to children aged 15–17 as well. In the other analyses to examine hidden CDWs, thresholds of 28 h or more and 35 h or more were applied to household survey data for Uganda and Paraguay, respectively [24]. In this study, we considered 21 h as the minimum to detect hidden CDWs in the Zimbabwean context, understanding that CDWs may not be working full-time and that they may be combining being a CDW with other forms of work or schooling [3,25]. We also included children declared as ‘live-in servants’ in the household roster—while these children are not formally ‘hidden’ CDWs, the small number of these children precluded distinguishing them separately.

In summary, the CDW variable included:

- Children who were classified as nieces/nephews, adopted/foster/stepchild, another relative, other (not related), uncles/aunts in the household roster, and,
- Doing 21 h or more of household chores, including hours spent fetching water or firewood, or,
- Children are declared as ‘live-in servant’ on the household roster (regardless of the number of hours spent on household chores, fetching water or firewood).

##### Biological Children

Biological children were categorised to include those children closely related to the household head/or spouse. These include sons, daughters, grandchildren, brothers, sisters, brothers-in-law, and sisters-in-law. While kinship care can encompass grandparental care [6], we opted to include grandchildren in this category owing to the closer biological relationship than more distant relatives, particularly in Zimbabwe where HIV/AIDs and out-migration have seen large numbers of children losing both parents and living with grandparents [26,27]. Furthermore, in the absence of grandparents, older siblings opt to raise their younger brothers and sisters, preferring to stay together as a close family unit connected by biological ties [27]. Additionally, cultural practices in Zimbabwe regard older siblings as primary caregivers in the absence of parents, resulting in sisters, brothers, brothers-in-law, and sisters-in-law being raised as biological children within such family setups [28].

##### Married Children

Married children included female household members who were below the age of 18 and in situations of marriage. In the household roster, these included spouses and daughters-in-law of the household heads. This relationship category was used to capture children who were living within a marriage setup in the respective households. Married boys were excluded from this category, given the small numbers (*n* = 5) in the data and sex-disaggregated analysis which follows.

##### Other Children

These were children likely to be in kinship care within the household but involved in less than 21 h of domestic chores including collecting firewood and fetching water. Other children included nieces/nephews, adopted/foster/stepchild, another relative, others (not related), and uncles/aunts in the household roster who were not classified as child domestic workers.

#### 2.2.2. Child Labour

We used the ILO age-specific definitions of child labour to identify children engaging in child labour: age 5 to 11 years, at least 1 h of economic activities or 21 h of unpaid household services per week; age 12 to 14 years, at least 14 h of economic activities or 21 h of unpaid household services per week, and age 15 to 17 years, at least 43 h of economic activities, with no threshold for several hours of unpaid household services. Economic activities in the MICS are classified as the following: (a) working on a plot, farm, food garden or looking after animals; (b) helping in family/relative’s business or running own business, (c) producing or selling articles, handicrafts, clothes, food or agricultural products, or (d) any other activity in return for income in cash or kind. In theory, CDWs could be declared as such under-economics activities in the other (d) category of activities in cash or kind, in the child labour MICS module. However, given that we do not know the specific activities here, these data were not used to create the CDW category above. Legislation in Zimbabwe categorises child labour differently from the ILO standard definitions. For instance, three major variations were introduced, namely: (a) a cut-off of 21 h or more per week about economic activities; (b) provision to allow for the involvement of children aged 15 years and above in some form of work as per national law, and (c) a cut-off of 35 h or more per week for children involved in unpaid care activities as constituting non-economic child labour [29].

#### 2.2.3. Hazardous Work

Hazardous child labour is the work that, by its nature or the circumstances in which it is carried out, is likely to harm the health, safety, or morals of children [30]. We used questions in the MICS dataset that asks if a child has been involved in any of the following: carrying heavy loads, working with dangerous tools or equipment, being exposed to dust or flames, exposed to extreme heat or temperature, exposed to loud noise or vibrations, working at heights, working with chemicals or explosives, and exposed to other unhealthy processes or activities. Any child involved in at least one of those conditions during the past week was regarded as exposed to hazardous child labour. Unlike in the ILO global estimates, hazardous work in the Zimbabwe MICS report did not also include children engaged in economic activities, household chores at age-specific thresholds considered hazardous, or children working under difficult conditions such as long hours or at night in the premises of an employer.

#### 2.2.4. Other Variables

The child’s socio-demographic and household characteristics used to describe the sample include, child’s sex (male, female) and age (5–19 years, 10–14 years, and 15–17 years), child orphanhood status, household wealth status (poorest, poorer, middle, richer, and richest), sex of household head (male, female), household head’s level of education (no education, primary, secondary, and higher), and religion (Catholic, Protestant, Pentecostal, Apostolic, traditional other religions, and none). Zimbabwe is home to numerous religions with most people following the Indigenous Christian Apostolic conservative sects [31]. Place of residence (rural or urban), and region or province were also used to describe the sample. Administratively, Zimbabwe has been divided into ten regions or provinces.

### 2.3. Data Analysis

We used descriptive statistics to calculate the prevalence of exposure to child labour, hazardous labour, disability, and other outcomes for each child group (CDW, married children, biological children, other children), and total for the whole population. Survey weights in the MICS were used to generate nationally representative prevalence estimates.

**Figure 1:**
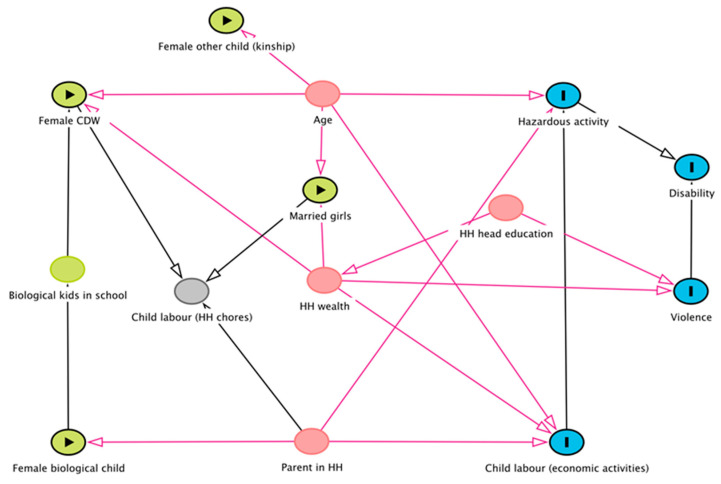
Directed Acyclic Graph of child categories and outcomes.

In the second stage of analysis, we used bivariable logistic regression to describe the frequency and distribution of the main exposure variables and each outcome variable (disability, hazardous work, any violence, child labour in economic activities). We did not include child labour in household chores as an outcome because hours in household chores was used to create the female CDW and other children’s categories. Next, we drew Directed Acyclic Graphs (DAGs) in DAGitty software [32] to map our conceptual assumptions and inform the selection of covariates in multivariable logistic regression models (Figure 1). DAGs were used to identify exposures for which we could estimate associations with the outcomes with minimal bias and thus include as covariates in statistical models [33]. The initial selection of covariates was based on literature regarding health outcomes and working conditions of CDWs, child brides, and children in kinship care. We selected covariates based on DAG recommended minimal adjustment sets, selecting the most parsimonious adjustment set [32]. As a result, eight separate multivariable analysis models representing each primary exposure were run to determine its effect on the outcome variables. Adjusted and unadjusted odds ratios (AOR and UOR) (with 95% confidence intervals (95% CI) were calculated using Stata Version 14 (Stata Corp, College Station, TX, USA).

## 3. Results

### 3.1. Main Characteristics of the Sample

Table 1 shows the percentage distribution of the sampled children by background characteristics. A total of 15,023 children were included in the sample, 48.9% of whom were girls and 17.7% were aged between 15 and 19 years. The mean age among the children was 10.3 years (SD, 3.7), which was highest among married children (15.4 years) and lowest among biological children (10.1 years). Nearly three-quarters of the children were living in households in rural areas and 16.7% had lost both parents. Almost a quarter of the children (22.8%) belonged to the poorest households while almost half of the households (42.3%) were followers of Indigenous Apostolic Christian sects.

Overall, the prevalence of child domestic work was 1.5% and the mean age of CDWs, 14.3 years was more than the mean age of all children (10.3 years) and biological children (10 years). The prevalence of children in situations of marriage and other children in kinship care arrangements was 0.5% and 11%, respectively. Girls and children aged 15–19 years constituted most of the CDWs, 56.4% and 55.7%, respectively. Compared to biological children, CDWs were more likely to be girls and much older (56.4% vs. 48.5% and 55.7% vs. 15.9%, respectively). Most CDWs (78.8%) were living in rural areas and rural provinces which include Mashonaland East, Mashonaland West, and Matabeleland North, and Midlands had more than half (57.9%) of CDWs. Orphanhood (the death of one or both parents) was more common among CDWs and married children than for biological children. Half of the CDWs (50.2%) and nearly half of married children (44.5%) were orphans while only 13.9% of biological children had lost both parents. CDWs were in wealthier households, where household heads were more educated. More than half (57.3%) of the CDWs were living in households where the head had attained secondary education or more while approximately half (42%) were from richer and richest households and a quarter were from households in the middle quintile. In contrast, married children were from poorer households, since 34.5% and 21.8% of married children were from the poorest and poorer households.

### 3.2. Children’s Living Arrangement

Table 2 shows the living arrangements of children in the sample study. Most children (38.6%) were living with both parents, and among those living with a single parent, women (26.5%) were more likely to live with children compared to single fathers (3.3%). Almost a third of the children (30.5%) were living with other relatives while only 1% were living with non-relatives. Compared with other child categories, CDWs had the highest proportion of children living with non-relatives (45.3%) and no CDWs lived with both parents. Most married children were living with other relatives (96.9%), showing that married girls moved in to live with either mothers- or fathers-in-law. While the proportion of biological children living with other relatives was a quarter (24.7%), it was more than a third for CDWs. Furthermore, CDWs living in another third-party household with their mother constituted 14.2% while those who moved into another household with their father were just 1%.

### 3.3. Children’s Engagement in Child Labour

Table 3 shows the proportion of children engaged in different types of child labour activities. More than a third (35%) of all children were engaged in different forms of child labour activities. The most common form of child labour was engaging in economic activities (25.5%), while hazardous work was 13.0% and 4.4% of children were in situations of child labour due to household chores. Higher proportions of boys rather than girls were engaged in any child labour activities (40.8% vs. 29.2%) and economic activities (31.7% vs. 19.0%). On the other hand, 3 times the proportion of girls were engaged in child labour due to household chores compared to boys (6.5% vs. 2.3%). Nearly half (47.9%) of married children and 35.9% of biological children were engaged in any child labour activity. Across the categories, CDWs were more likely to be engaged in child labour activities. For instance, the proportion of CDWs engaged in economic activities was 31.7% while it was 4.6% for married children. Higher proportions of married children (29.5%) followed by CDWs (19.3%) and biological children (13.2%) were engaged in hazardous work. With respect to gender, higher proportions of boys rather than girls across all the child categories were engaged in child labour activities, except for household chores. Over twice the proportion of male CDWs was engaged in economic activities compared to their female counterparts (45.7% vs. 20.8%). Similarly, among other children, a fifth of boys (21.4%) and a tenth (12.0%) of girls were engaged in economic activities. Higher proportions of boys were involved in hazardous work across all the child categories, particularly among CDWs. A third (34.8%) of boy CDWs compared to just 7.3% of girl CDWs were exposed to child labour in hazardous work, while among biological children, it was 15.6% for boys and 10.5% for girls.

### 3.4. Children’s Engagement in Economic Activities

Table 4 shows the proportion of children engaged in different types of economic activities. A quarter of children (25.5%) were engaged in economic activities, with a higher proportion of boys (31.7%) than girls (19.0%) involved in household economic activities. On average, children spent 4.9 h on economic activities in the past week. Boys and CDWs spent more time on economic activities compared to girls and other categories of children. CDWs spent 18.2 h on economic activities, while biological, married, and other children spent only 4.7, 6.8, and 4.6 h, respectively on economic activities. Comparing within CDWs, boys were almost 3 times more likely to be engaged in economic activities than girls. Gardening is the main economic activity undertaken by most children. Nearly half of all children (48.4%) reported working in the garden, with boys more likely to be engaged in this form of economic activity (56.3%) than girls (40.1%). Working on family farms was more common among married children and CDWs. Nearly two-thirds of both CDWs and married children were working on family farms. Across all the child categories, boys were involved more in family farming, with the highest proportion of 85.3% among male CDWs. On the contrary, working in the family business was uniform across all children except for CDWs, where nearly twice the proportion of girls than boys were involved in this type of activity. There were wide variations in children’s engagement in other activities in exchange for payment in cash or kind. While the average for all children was 3.2%, it was more than 10 times (34.3%) for CDWs and 3 times more (9.5%) for married children. Three times the proportion of male CDWs were involved in other activities compared to female CDWs.

### 3.5. Children’s Engagement in Household Tasks

Table 5 shows the proportion of children engaged in different household tasks including fetching water and collecting firewood. Children spent, on average, 5.9 h on household chores with girls spending more hours (7.6) compared to boys (4.2) on domestic duties. Biological children spent the least time doing household chores (5.6 h), compared to 25 and 23 h for CDWs and married children, respectively (Please note that the hours variable was used to construct the CDW variable (with a cut-off of 21 h) so the average time spent on household chores will be higher among CDWs). Tasks such as cooking, washing dishes and clothes, and fetching water were common among the children. More than half of the children were involved in washing the dishes, cleaning the house, and fetching firewood. The majority of CDWs and married children were engaged in more household activities.

A large proportion (97%) of married children reported washing dishes or cleaning the house and 93% and 83% doing cooking and laundry activities, respectively. Similarly, 69% of CDWs reported washing dishes and clothes and cleaning the house. In contrast, about 50% of biological children reported washing dishes and clothes, cleaning the house, and fetching water. Female child domestic workers spent more hours on household tasks when compared to male child domestic workers Among CDWs, twice the proportion of girls washed clothes and cared for children, while 3 times the proportion cooked, washed dishes, and cared for the sick compared to boys. Similarly, collecting firewood and fetching water was gendered, with higher proportions of girls (24.7% and 59.8%) than boys engaging in these activities (17.4%, 47.9%) overall. Interestingly, among CDWs, the proportion of boys fetching water and collecting firewood is almost similar to those of girls (78% vs. 74.4% and 26.8% vs. 25.0%).

### 3.6. Children’s Exposure to Hazardous Work

Table 6 shows the proportion of children engaged in different forms of hazardous working conditions. Overall, 13% of children were engaged in hazardous working conditions. Working with dust, fumes, or gases and in extreme temperatures were the most common hazardous activities with proportions of 14.3% and 10.2%, respectively. The proportion of children engaged in hazardous working conditions was high among boys, CDWs and married children. Nearly a third (30%) of married children and a fifth (19.3%) of CDWs were involved in hazardous working conditions compared to just over a tenth (13.0%) of biological children. Among CDWs, 5 times the proportion of boys were involved in hazardous working conditions (34.8% vs. 7.3%) compared to girls. For instance, among CDWs, 14.8% were exposed to working with dust, fumes, and gases overall, with 3 times the proportion of boys (21.1%) exposed than girls (7.7%). Married children had the highest proportion of children carrying heavy loads (15.2%), working with dangerous tools (16.4%), working in extreme temperatures (18.2%), and being exposed to other hazards (16.9%).

### 3.7. Children’s Experiences of Functional Disabilities

Table 7 and Table 8 shows the proportion of children with functional difficulties. Ten percent of all children had some form of functional disability, with the highest proportion (14.2%) among CDWs and the lowest (7.2%) among married children. The proportion of child disability is higher among boys (11.1%) than girls (9.0%). However, among CDWs, the proportion is 4 times higher among girls than boys (21.3% vs. 5.0%). Moreover, 3 times the proportion of female CDWs had disabilities (21.3%) compared to married children (7.2%). More children reported learning, depression, remembering and anxiety difficulties than other types of difficulties. Learning and anxiety difficulties were higher among CDWs relative to other child categories, 4.7% and 5.1%, respectively, while among married children, anxiety was high (4.5%). Compared with biological children, twice the proportion of CDWs had difficulties with hearing, learning, making friends. and anxiety.

### 3.8. Children’s Experiences of Child Discipline

Table 9 shows the proportion of children who experience different forms of child discipline. Overall, child discipline was high among the sample (87.7%) and the proportion of boys and girls experiencing any form of child discipline were almost the same throughout the child categories. While, on average, the proportion of children experiencing physical punishment alone (38.8%) was the lowest. More than two-thirds (67.5%) of married children have experienced this form of discipline. Among CDWs, the proportion of children experiencing physical punishment was 4 times higher among girls (46.1%) than boys (11.9%). Psycho-aggression was high among married children since almost three-quarters (77.4%) have experienced this form of discipline.

### 3.9. Multivariable Analysis

The results from the multivariable analysis and associated Directed Acyclic Graphs are shown in Table 10. In the multivariable analysis, comparing the exposures among girls, the odds of having a functional disability were higher among girls in child domestic work when compared to biological girls. In the unadjusted model, child domestic workers had 2.8 times the odds of having a disability when compared to biological children (UOR 2.82, CI:1.27–6.27). Girls working as child domestic workers had nearly 3 times (1/0.35) the odds of having a functional disability when compared to girls living under kinship care (UOR0.35, 95% CI:0.16–0.79). In terms of the experience of child discipline among all children, the odds of exposure to any form of violent discipline were lower among children living in kinship care when compared to biological children of household heads (AOR 0.68, 95% CI:0.51–0.91).

The odds of being involved in child economic activities was higher among female child domestic workers when compared to female married children. In the unadjusted model, girls who were child domestic workers had 5.5 times the odds of being engaged in economic activities than married girls (UOR 5.52, CI:1.19–25.75). When comparing biological children with the other children’s categories, the odds of involvement in economic activities was higher among child domestic workers (AOR 2.28, 95% CI:1.18–4.40), and lower among children in kinship care (AOR 0.58, 95% CI:0.44–0.76) and married children (AOR 0.28; 95% CI:0.08–0.99).

Married girls had higher odds of exposure to hazardous working conditions compared to female child domestic workers (AOR 0.25, 95% CI:0.08–0.80). When compared to girls under kinship care, girls in child domestic work had 3.9 times the odds of being engaged in any form of hazardous activities (AOR 3.85, 95% CI:1.33–11.18). The odds of performing hazardous activities were high among biological girls when compared to female child domestic workers (AOR 0.26, 95% CI:0.09–0.75). In terms of hazardous work exposure among all children, the odds were higher among married children (AOR 2.76, 95% CI:1.26–6.02) and lower among children in kinship care (AOR 0.67, 95% CI:0.49–0.93). when compared to biological children.

## 4. Discussion

This study presented the first intersectional analysis to compare CDWs, married children, children in kinship care and those biologically related to household heads using nationally representative data in Zimbabwe. These groups of children are usually considered in isolation, but research into the linkages could be beneficial for identification and programming purposes (for example, household outreach for married children and CDWs will be similar, and both are particularly hidden groups of children). The study utilised the household roster to determine the child categories and all children who shared a biological relationship with the household such as grandchildren, brothers, and sisters were included under biological children. In Zimbabwe, it is common for older siblings to take care of their younger siblings and for grandparents to foster their grandchildren within a close family setup [26].

Our analysis shows that more girls than boys are engaged in child domestic work. This finding is supported by previous studies that show that child domestic work is a gendered phenomenon that disproportionately affects girls [34]. While estimates [35] show that child domestic workers tend to be young, our analysis shows that, like married children, most CDWs are aged 15–17 in Zimbabwe. Unlike child marriage, child domestic work is a hidden phenomenon that is difficult to tackle since most households do not disclose the presence of CDWs. In our analysis, most CDWs were in wealthier households where household heads were more educated. This may be a result of wealthier relatives taking in children to stay with them, and therefore, they end up doing domestic work in the household. On the other hand, the analysis confirms poverty as a driver of CDWs since most married children were in much poorer households [36]. The study also shows that more CDWs were living with non-relatives, and for those who had a parent in the household, it was likely to be a mother. This confirms that child domestic work is a poverty phenomenon that mainly follows a gender inter-generational pattern [37,38]. This may mean that, unlike men, women move in to work in other households together with their children who end up as CDWs. Elsewhere, younger CDWs are more likely to be living with relatives [39]. They may be more vulnerable to violence and less likely to leave due to their age and living under a kinship obligation [40].

Like previous studies, our findings show that apart from domestic work, CDWs are also engaged in more hazardous activities and economic labour activities [15,34]. CDWs may also be doing economic activities, especially within the context of household duties. In this study, a high proportion (62.4%) of CDWs were doing agricultural activities, for which they may or may not be paid (the question does not ask if children are paid in cash or kind). The phrasing of this question assumes that households are doing agricultural activities for economic gain, and does not consider subsistence agriculture, of which we would expect CDWs to partake in alongside their household chores—locally, in Zimbabwe, helping on the household plot, farm, food garden, or looking after animals could be considered as part of the tasks CDWs undertake. The study showed similarities in the activities performed by CDWs and married children. CDWs, especially boys, were working with chemicals, dust, fumes, and carrying heavy loads. This is probably linked to economic activities such as tobacco farming, timber logging, sugar cane production, and gold panning around Zimbabwe [41]. However, we do not have detailed occupational information so cannot distinguish when children are exposed to these hazards. Similarly, married girls were involved with carrying heavy loads, which may be from fetching water for household consumption or gardening activities, which is a major source of livelihood among women in Zimbabwe [42]. Other similarities between CDWs and married children were in terms of average hours spent on domestic tasks (24–25 h) and proportions involved in specific activities, for instance, working on family farms (62–64%). This may be linked to gender since we have observed also that female CDWs and married children were engaged in traditional domestic chores such as cooking, washing dishes, cleaning the house, or washing clothes.

Our findings show a higher proportion of CDWs (14.2%) experienced functional difficulty compared to lower proportions for biological children, other children, and married children. It is unclear whether functional difficulties would cause a child to be sent away as a CDW, or whether it is the result of engagement in being a CDW. More research is needed to explore the direction of this relationship. When we compare with married children, they tend to report less functional difficulties than CDWs, probably because when choosing a child bride, children with functional disabilities are less likely to be chosen. Depression was low across all groups of children, maybe because it is a poorly understood concept among caregivers reporting on their behalf, and among children themselves. Generally, as we start to document the health impacts of CDW, we will be able to compare with the health impacts of child marriage [43] and systematically compare harms. Household surveys and research should ask whether girls are married (and when), whether they are a CDW (in other people’s homes), and where they are experiencing violence, otherwise, CDWs might be missed. For instance, in one qualitative study, half of the child brides in the sample were working as CDWs in other homes, but only husband-perpetrated IPV was explored, and not, for example, violence experienced as a CDW from employers [44]. Similarly, there is no explicit connection between the literature on the health of children left behind by migrating parents [45] who are usually left in kinship care, and examination of how differential living arrangements of children left behind can affect health outcomes. Similar to previous studies [46], child discipline was high across all groups of children, but particularly for married children—suggesting a high prevalence of IPV for this group [47]. Similarly, more intersectional analyses should be conducted of existing household surveys (MICS, Demographic and Health Surveys (DHS)) with information on living arrangements, child marriage, and child labour, to explore similarities and differences between these vulnerable groups of children, which, as our paper demonstrates, are not mutually exclusive categories. Recently, the DHS has added a vulnerable children module [48], but until we start to conceptualise and demonstrate the links, programming for children will continue to be disjointed.

The major strength of this study was that nationally representative MICS data were used. The MICS survey employs standardised data collection protocols administered by trained study personnel with standardised measurement equipment using validated questionnaires. Nonetheless, some study limitations were also observed. Since secondary data were used, we relied on the responses of the child’s primary caregiver. Given the hidden nature of child domestic workers, some caregivers might both fully disclose the nature of relationships, as well as the extent of children engaged in labour activities. Information on other important subjective wellbeing factors such as depression and experience of violence are not easily measured within household surveys especially when a child is being abused by the caregiver. We did not conduct multilevel analysis to compare CDWs with children in other categories in the same households as this was not possible due to the design of the MICS surveys, whereby one child is randomly selected for the detailed child questionnaire with child labour, child discipline, and functioning. Multi-level modelling could have helped in explaining the differences between CDWs and other children living in the same household. While studies have examined differences between CDWs and neighbourhood controls [25,40,49], it is unclear whether CDWs and controls are in the same or different households. Quantitative analysis which compares conditions and outcomes of CDWs and biological children in the same household would enable an analysis of how CDWs and biological children are treated differently [21]. For other children, we do not know what proportion of non-relative close family friends are known to the child—therefore, we cannot be definitive in assertions that these children are in kinship care. Similarly, for ease of analysis and according to closer relationship ties in Zimbabwe between grandparents and grandchildren, we included those children in the biological child category when they could be considered as ‘Other children’ likely to be in kinship care.

## 5. Conclusions

We have provided the first intersectional analysis to compare CDWs, married children, children in kinship care, and those biologically related to household heads using nationally representative data. CDWs are among the hardest to reach and least visible groups of child labourers, alongside married children, who worked similarly long hours as CDWs but had greater exposure to hazardous work. Notably, our study showed that female CDWs more frequently had functional disabilities. This research could be repeated in other settings to design targeted household interventions that could reach both hidden groups of children. Typically, CDWs and married children are not considered in child protection systems which tend to prioritise abuse, neglect, and separation of biological children. Our findings show that these dually related forms of child work should be given equal attention in policy and programmatic interventions, to safeguard the rights of children in marriages and expose hidden forms of domestic work.

## Figures and Tables

**Table 1:** Characteristics of Child Domestic Workers, Biological children, Married children, and other children in households in Zimbabwe, 2019 (*N* = 15,023, %).

Variables	Child Domestic Worker	Biological Children	Married Children	Other Children	All Children
**Prevalence** **Mean Age (SD)**	1.5 14.3 (2.7)	87.0 10.1 (3.6)	0.5 15.4 (2.7)	11.0 11.1 (3.7)	100.0 10.3 (3.7)
**Age Group**					
5–9	7.3	48.1	3.1	33.9	46.0
10–14	40.0	36.0	18.0	40.0	34.4
15–19	55.7	15.9	78.9	26.1	17.7
**Child Sex**					
Male	43.4	51.5	-	51.6	51.1
Female	56.4	48.5	100.0	48.4	48.9
**Household Wealth**					
Poorest	12.8	23.6	34.5	34.5	22.8
Poorer	20.0	23.2	21.8	21.8	22.5
Middle	25.4	20.4	17.4	17.4	20.7
Richer	24.1	16.8	18.4	18.4	17.5
Richest	17.7	16.0	7.8	7.8	16.4
**Household Head Education**					
No Education	3.9	6.4	11.9	3.8	6.1
Primary	39.5	41.0	42.3	32.3	40.0
Secondary	41.5	44.0	42.1	51.8	44.8
Higher	15.8	8.6	3.8	12.2	9.1
**Household Head Religion**					
Catholic	9.8	6.0	3.9	8.0	6.3
Protestant	14.6	15.3	7.0	18.5	15.6
Pentecostal	26.1	14.2	9.2	15.0	14.4
Apostolic	29.4	43.2	46.2	36.9	42.3
Other Religion	6.2	3.5	15.3	5.6	3.8
Traditional	1.3	4.4	5.1	4.1	4.3
No religion	12.7	13.4	13.3	11.9	13.3
**Place of Residence**					
Urban	21.2	25.7	25.3	31.7	26.3
Rural	78.8	74.3	74.7	68.3	73.7
**Region of Residence**					
Manicaland	4.1	4.1	3.3	6.7	4.3
Mashonaland Central	10.0	16.4	11.2	11.1	15.7
Mashonaland East	13.3	9.6	27.4	7.9	9.6
Mashonaland West	10.3	10.9	7.5	9.3	10.7
Matabeleland North	21.4	11.7	10.3	13.4	12.0
Matabeleland South	9.1	6.7	4.5	5.4	6.1
Midlands	5.0	5.8	3.6	11.2	6.4
Masvingo	12.9	11.4	9.9	12.6	11.5
Harare	7.9	12.2	9.5	11.6	12.0
Bulawayo	6.0	11.8	12.9	11.1	11.7
**Orphanhood Status**					
No	49.8	86.1	55.5	67.0	83.3
Yes	50.2	13.9	44.5	33.0	16.7
**Number**	**225**	**13,065**	**86**	**1647**	**15,023**

Bold for prevalence and Mean Age group.

**Table 2:** Living Arrangements of Child Domestic Workers, Biological children, Married children, and other children in households in Zimbabwe, 2019 (*N* = 15,023).

Living with *	Child Domestic Worker	Biological Children	Married Children	Other Children	All Children
**Only Mother**	14.2	27.5	0.0	14.8	26.5
**Only Father**	1.0	3.7	0.9	0.4	3.3
**Both Parents**	0.0	44.1	2.1	2.0	38.6
**Other Relative**	39.4	24.7	96.9	72.6	30.5
**Non-Relative**	45.3	0.0	0.0	2.9	1.0
**Total**	100.0	100.0	100.0	100.0	100.0
**Number**	**225**	**13,065**	**86**	**1647**	**15,023**

* Parental living arrangements were asked in a separate question to relationship to household head: percentages sum to >100%.

**Table 3:** Proportion of Child Domestic Workers, Biological children, Married children, and other children in situations of child labour (*N* = 15,023, %).

Child Labour	Child Domestic Worker			Biological Children			Married Children			Other Children			All Children		
	M	F	T	M	F	T	M	F	T	M	F	T	M	F	T
**Economic Activities**	45.7	20.8	31.7	32.7	20.1	26.6	-	4.6	4.6	21.4	12.0	16,8	31.7	19.0	25.5
**Household Chores**	21.4	65.6	46.3	2.3	5.8	4.0	-	35.2	35.2	0.0	0.0	0.0	2.3	6.5	4.4
Hazardous Work *	34.8	7.3	19.3	15.6	10.5	13.2	-	29.5	29.5	11.9	6.8	9.3	15.4	10.3	13.0
**Any Child Labour**	79.1	74.8	76.7	41.7	29.7	35.9	-	47.9	47.9	28.9	15.6	22.0	40.8	29.2	35.0
**Number**	**98**	**127**	**225**	**6727**	**6338**	**13,065**	**-**	**86**	**86**	**850**	**797**	**1647**	**7675**	**7348**	**15,023**

* only includes any hazardous activities and not hours of work above age-specific thresholds (see MICS report).

**Table 4:** Proportion of children engaged in different economic activities and average hours spent by sex (*N* = 15,023, %).

Economic Activity	Child Domestic Worker			Biological Children			Married Children			Other Children			All Children		
	M	F	T	M	F	T	M	F	T	M	F	T	M	F	T
**Family Farm**	85.3	44.8	62.4	56.5	40.9	48.9	-	64.8	64.8	51.6	30.6	41.5	56.3	40.1	48.4
**Family Business**	4.0	7.1	5.7	3.1	3.0	3.1	-	3.0	3.0	3.3	2.4	2.9	3.1	3.0	3.1
**Sold Articles**	0.5	3.6	0.8	1.3	0.9	1.4	-	6.6	6.6	0.6	1.1	0.8	1.2	1.0	1.1
**Other Activity**	54.3	18.7	34.3	3.2	2.1	2.7	-	9.5	9.5	3.9	2.4	3.2	3.9	2.5	3.2
**Total**	45.7	20.8	31.7	32.7	20.1	26.6	-	4.6	4.6	21.4	12.0	16,8	31.7	19.0	25.5
**Average Hours**	30.0	9.1	18.2	6.3	3.0	4.7	-	6.8	6.8	6.8	2.2	4.6	6.6	3.0	4.9
**Number**	**98**	**127**	**225**	**6727**	**6338**	**13,065**	**-**	**86**	**86**	**850**	**797**	**1647**	**7675**	**7348**	**15,023**

**Table 5:** Proportion of Children engaged in household tasks by sex (*N* = 15,023, %).

Household Tasks	Child Domestic Worker			Biological Children			Married Children			Other Children			All Children		
	M	F	T	M	F	T	M	F	T	M	F	T	M	F	T
**Shopping for household**	22.4	40.0	32.2	25.4	24.8	25.1	-	31.3	31.3	27.2	30.2	28.7	25.6	25.7	25.7
**Cooking**	33.4	81.3	60.4	22.4	41.6	31.7	-	83.1	83.1	17.4	46.0	31.3	21.9	43.2	32.4
**Washing dishes or cleaning the house**	36.9	94.7	69.5	38.7	75.1	56.4	-	97.9	97.9	34.2	78.8	55.8	38.2	76.1	56.8
**Washing clothes**	43.9	88.6	69.1	28.4	46.7	37.3	-	93.4	93.4	38.1	58.8	48.1	29.6	49.3	39.3
**Caring for children**	25.1	64.1	47.1	20.6	33.1	26.7	-	45.2	45.2	18.6	30.5	24.3	20.4	33.5	26.8
**Caring for old or sick**	0.6	2.6	1.7	1.5	2.9	2.2	-	3.2	3.2	1.2	4.8	2.9	1.5	3.1	2.3
**Fetching water**	78.0	74.4	76.5	47.1	59.5	53.1	-	91.0	91.0	50.3	56.8	53.4	47.9	59.8	53.7
**Collecting firewood**	26.8	25.0	25.8	17.7	24.8	21.2	-	39.0	39.0	13.8	22.5	18.0	17.4	24.7	21.0
**Other domestic tasks**	20.2	21.0	20.7	11.7	14.0	12.6	-	46.8	46.8	11.1	14.3	12.6	11.7	14.3	13.0
**Average hours (water, firewood + chores)**	15.8	32.1	25.0	4.2	7.2	5.6	-	23.9	23.9	3.0	5.0	4.0	4.2	7.6	5.9
**Number**	**98**	**127**	**255**	**6727**	**6338**	**13,065**	**-**	**86**	**86**	**850**	**797**	**1647**	**7675**	**7348**	**15,023**

**Table 6:** Proportion of Children engaged in Hazardous working conditions (*N* = 15,023, %).

Hazardous Activity	Child Domestic Worker			Biological Children			Married Children			Other Children			All Children		
	M	F	T	M	F	T	M	F	T	M	F	T	M	F	T
**Heavy loads**	11.9	0.7	6.7	6.1	5.4	5.8	-	15.2	15.2	5.3	3.3	4.6	6.2	15.2	5.8
**Working with dangerous tools**	3.1	4.5	3.7	3.7	5.1	4.3	-	16.4	16.4	4.3	2.9	3.8	3.8	5.2	4.3
**Dust, fumes or gas**	21.1	7.7	14.8	9.9	11.3	10.4	-	11.5	11.5	5.4	8.3	6.5	9.6	10.9	10.2
**Extreme temperature**	11.6	5.3	8.6	16.5	12.3	14.8	-	18.2	18.2	12.2	9.6	11.2	15.9	12.0	14.3
**Loud noise or vibration**	0.0	0.8	0.4	0.7	0.9	0.8	-	0.0	0.0	0.8	0.7	0.7	0.7	0.9	0.7
**Work at heights**	1.1	0.0	0.6	0.8	0.5	0.6	-	0.0	0.0	0.3	0.0	0.2	0.7	0.4	0.6
**Work with chemicals/explosives**	13.7	3.3	8.8	2.1	1.0	1.6	-	0.0	0.0	1.0	0.7	0.9	2.2	1.0	1.7
**Other hazard**	11.5	3.3	7.6	4.5	2.1	3.5	-	16.9	16.9	1.0	0.2	1.3	4.4	2.3	3.5
Any hazard *	34.8	7.3	19.3	15.6	10.6	13.2	-	29.5	29.5	11.9	6.7	9.3	15.4	10.3	13.0
**Number**	**98**	**127**	**225**	**6727**	**6338**	**13,065**	**-**	**86**	**86**	**850**	**797**	**1647**	**7675**	**7348**	**15,023**

* only includes hazardous activities and not hours of work above age-specific thresholds (see MICS report).

**Table 7:** Proportion of children with Functional Difficulties (*N* = 15,023, %).

Functional Domain	Child Domestic Worker	Biological Children	Married Children	Other Children	All Children
**Seeing**	0.0	0.7	0.0	0.3	0.7
**Hearing**	1.4	0.5	0.0	0.3	0.5
**Walking**	0.0	0.1	0.0	0.0	0.1
**Communication**	0.2	0.5	0.0	0.8	0.5
**Selfcare**	0.0	0.5	0.0	0.5	0.5
**Learning**	4.7	2.5	0.0	1.7	2.4
**Remembering**	5.9	1.8	0.7	1.5	1.8
**Concentrating**	0.0	2.0	0.0	1.1	1.9
**Accepting Change**	0.0	1.0	0.0	1.0	1.0
**Behaviour**	0.0	1.7	0.7	1.0	1.6
**Making Friends**	2.8	0.6	2.1	0.7	0.7
**Depression**	0.9	1.8	0.0	2.4	1.9
**Anxiety**	5.1	2.3	4.5	3.9	2.5
**Any Functional Difficulty**	14.2	10.1	7.2	9.7	10.1
**Number**	**225**	**13,065**	**86**	**1647**	**15,023**

**Table 8:** Proportion of children with Functional Difficulties by sex (*N* = 15,023, %).

Discipline	Child Domestic Worker			Biological Children			Married Children			Other Children			All Children		
	M	F	T	M	F	T	M	F	T	M	F	T	M	F	T
**Any Functional Difficulty**	5.0	21.3	14.2	11.3	8.8	10.1	-	7.2	7.2	9.8	9.6	9.7	11.1	9.0	10.1
**Number**	**98**	**127**	**225**	**6727**	**6338**	**13,065**	**-**	**86**	**86**	**850**	**797**	**1647**	**7675**	**7348**	**15,023**

**Table 9:** Proportion of children with Functional Difficulties by sex (*N* = 15,023, %).

Discipline	Child Domestic Worker			Biological Children			Married Children			Other Children			All Children		
	M	F	T	M	F	T	M	F	T	M	F	T	M	F	T
**Psycho Aggression**	60.2	52.5	47.8	59.0	56.9	58.0	-	77.4	77.4	48.3	47.3	47.8	57.9	77.4	56.9
**Physical Punishment**	11.9	46.1	29.6	39.4	38.2	38.8	-	67.5	67.5	28.6	28.7	28.7	38.1	37.4	38.8
**Non-Violent**	80.6	52.6	34.0	59.2	56.8	42.0	-	30.5	30.5	49.3	47.9	51.4	58.4	55.9	42.9
**Any Child Discipline**	92.9	95.5	94.3	89.9	86.2	88.1	-	77.4	77.4	83.1	85.5	84.3	89.3	86.2	87.7
**Number**	**48**	**52**	**99**	**5591**	**5395**	**10,987**	**-**	**18**	**18**	**638**	**629**	**1267**	**6277**	**6094**	**12,371**

**Table 10:** Child categories associated with disability, violence, Child Labour (economic activities) and hazardous work (*N* = 12,371).

	Any Functional Disability		Any Violence		Child Economic Labour		Any Hazardous Activity	
Primary Exposure	UOR	AOR	UOR	AOR	UOR	AOR	UOR	AOR
Biological Girls **^1^**	2.816 * (1.265–6.269)	2.651 (0.685–10.27) ^a^	3.429 (0.698–16.84)	3.863 (0.790–18.88) ^b^	1.048 (0.464–2.367)	1.628 (0.693–3.824) ^c^	0.657 (0.246–1.754)	0.260 * (0.0895–0.753) ^d^
Kinship Care Girls **^2^**	0.355 * (0.160–0.790)	0.377 (0.0974–1.461) ^a^	0.292 (0.0594–1.432)	0.259 (0.053–1.266) ^b^	0.954 (0.423–2.156)	0.614 (0.262–1.442) ^c^	1.521 (0.570–4.061)	3.852 * (1.328–11.18) ^d^
Married Girls **^3^**	3.474 (0.861–14.03)	3.939 (0.904–17.17) ^a^	-	-	5.524 * (1.185–25.75)	5.884 (0.849–40.79) ^c^	0.186 ** (0.0574–0.606)	0.254 * (0.0802–0.804) ^d^
Children (Ref: Bio-Children) **^4^**								
Child Domestic Workers	1.483 (0.729–3.017)	1.612 (0.784–3.314)	2.224 (0.809–6.116)	2.353 (0.855–6.477)	1.281 (0.723–2.271)	2.276 * (1.176–4.404)	1.571 (0.830–2.976)	1.064 (0.534–2.123)
Other Kinship Children	0.935 (0.680–1.285)	0.993 (0.718–1.371)	0.723 * (0.546–0.959)	0.677 ** (0.506–0.905)	0.560 *** (0.431–0.727)	0.576 *** (0.435–0.762)	0.678 * (0.499–0.923)	0.674 * (0.487–0.933)
Married Children	0.697 (0.226–2.155)	0.720 (0.234–2.214)	0.463 (0.0678–3.157)	0.443 (0.0706–2.776)	0.132 ** (0.0365–0.475)	0.280 * (0.079–0.996)	2.758 * (1.263–6.023)	1.534 (0.719–3.274)

Notes: OR: odds ratio, *** *p* < 0.001, ** *p* < 0.01, * *p* < 0.05. **^1^** This model includes female biological children and female child domestic workers. The reference category for this primary exposure is female child biological children. **^2^** This model includes other female children in kinship care and female child domestic workers. The reference category for this primary exposure is female children in kinship care. **^3^** This model includes female married children and female child domestic workers. The reference category for this primary exposure is female married children. **^4^** This model includes all children (male and female), and biological children is the reference category. Disability model adjusted for hazardous work, child discipline, sex; any violence model adjusted for household education, household wealth, sex; child economic labour model adjusted for age, sex, household wealth, parent in household; hazardous activity model adjusted for age, sex, household wealth, parent in household. ^a^ Adjusted for hazardous work, violence, sex. ^b^ Adjusted for household wealth, sex. ^c^ Adjusted for age, household wealth, parent in household, sex. ^d^ Adjusted for age, child labour (economic activities), parent in household, sex.

## Data Availability

The data presented in this study are openly available at https://mics.unicef.org/, accessed on 23 May 2021.
